# High-parameter phenotypic characterization reveals a subset of human Th17 cells that preferentially produce IL-17 against *M. tuberculosis* antigen

**DOI:** 10.3389/fimmu.2024.1378040

**Published:** 2024-04-18

**Authors:** Paul Ogongo, Anthony Tran, Florence Marzan, David Gingrich, Melissa Krone, Francesca Aweeka, Cecilia S. Lindestam Arlehamn, Jeffrey N. Martin, Steven G. Deeks, Peter W. Hunt, Joel D. Ernst

**Affiliations:** ^1^ Division of Experimental Medicine, University of California, San Francisco, San Francisco, CA, United States; ^2^ Department of Tropical and Infectious Diseases, Institute of Primate Research, Nairobi, Kenya; ^3^ Drug Research Unit, Department of Clinical Pharmacy, School of Pharmacy, University of California, San Francisco, San Francisco, CA, United States; ^4^ Department of Epidemiology and Biostatistics, University of California, San Francisco, San Francisco, CA, United States; ^5^ Center for Infectious Disease and Vaccine Research, La Jolla Institute for Immunology, La Jolla, CA, United States; ^6^ Division of HIV, Infectious Diseases, and Global Medicine, University of California, San Francisco, San Francisco, CA, United States

**Keywords:** interleukin-17, CD4 T-cells, antigen-responsive, immunity, tuberculosis, ART-suppressed, HIV, kynurenine pathway

## Abstract

**Background:**

Interleukin-17–producing CD4 T cells contribute to the control of *Mycobacterium tuberculosis* (*Mtb*) infection in humans; whether infection with human immunodeficiency virus (HIV) disproportionately affects distinct Th17-cell subsets that respond to *Mtb* is incompletely defined.

**Methods:**

We performed high-definition characterization of circulating *Mtb*-specific Th17 cells by spectral flow cytometry in people with latent TB and treated HIV (HIV-ART). We also measured kynurenine pathway activity by liquid chromatography-mass spectrometry (LC/MS) on plasma and tested the hypothesis that tryptophan catabolism influences Th17-cell frequencies in this context.

**Results:**

We identified two subsets of Th17 cells: subset 1 defined as CD4^+^Vα7.2^−^CD161^+^CD26^+^and subset 2 defined as CD4^+^Vα7.2^−^CCR6^+^CXCR3^−^cells of which subset 1 was significantly reduced in latent tuberculosis infection (LTBI) with HIV-ART, yet *Mtb*-responsive IL-17–producing CD4 T cells were preserved; we found that IL-17–producing CD4 T cells dominate the response to *Mtb* antigen but not cytomegalovirus (CMV) antigen or staphylococcal enterotoxin B (SEB), and tryptophan catabolism negatively correlates with both subset 1 and subset 2 Th17-cell frequencies.

**Conclusions:**

We found differential effects of ART-suppressed HIV on distinct subsets of Th17 cells, that IL-17–producing CD4 T cells dominate responses to *Mtb* but not CMV antigen or SEB, and that kynurenine pathway activity is associated with decreases of circulating Th17 cells that may contribute to tuberculosis immunity.

## 1 Introduction

Increasing evidence indicates that CD4 T cells that produce interleukin-17 (IL-17), broadly termed T helper 17 (Th17) cells, contribute to the control of TB. In *Mtb*-infected adolescents, IL-17 transcriptional signatures decreased in the blood of those who progressed to active TB compared to non-progressors (1); another study found a subset of CD4 T cells that produce IL-17 in response to *Mtb* antigens that is less abundant in TB progressors than in non-progressors (2). *Mtb*-responsive CD4 T cells producing IL-17 are enriched in human lungs compared to matched blood and inversely correlated with plasma IL-1β, suggesting a role in the control of *Mtb*(3). IL-17–producing CD4 T cells are depleted in human immunodeficiency virus (HIV) infection and their depletion contributes to the progression to AIDS through breakdown in intestinal mucosal barrier function reviewed in (4, 5), in mechanisms that depend in part on the expression of CCR5 by IL-17–producing CD4 T cells.

Several approaches are used to identify T helper 17 cells (herein denoted “Th17” cells to include all subsets regardless of criteria used). IL-17 production upon stimulation is the canonical signature of Th17 cells (6, 7). Although this identifies Th17 cells, it does not classify all cells in the Th17 lineage, and other cells can produce IL-17 (8–11). In the blood of healthy donors and synovial fluid of rheumatoid arthritis patients, a CCR6^+^subset contained all IL-17–producing T cells expressing *RORC* mRNA (12), the transcription factor that supports Th17 differentiation in humans. The use of CCR6 as a marker of Th17 cells has been replicated in naïve cord blood (13), inflammatory diseases (14), and infections like TB (15). The cell surface markers CD26 (16–19) and CD161 (20–23) have also been used to identify Th17 cells. CD26^hi^CD4^+^ T cells were demonstrated to co-express CD161 and CCR6 and to be enriched for the production of IL-17 (16). In transcriptomic studies, Th17 cells are identified by expression of *RORC*, *IL23R* (supports the expansion of IL-17–producing cells) and *IL-17* mRNA.

Host metabolism impacts immune responses (24), including by driving cell differentiation. One example is the kynurenine pathway of tryptophan catabolism whose products, collectively called kynurenines, inhibit Th17 while promoting Treg development (25–28). In TB, circulating tryptophan (Trp) concentrations decline in persons with latent tuberculosis infection (LTBI) progressing to active TB (29) and Trp concentration increases with TB treatment (25). Similarly, HIV-infected persons have low-circulating Trp concentration with a link to pathogenesis (30). Plasma kynurenine/tryptophan ratios (K/T) can distinguish humans with active TB from those without and are significantly higher in multi-drug resistant (MDR-TB) patients with or without HIV coinfection compared to controls (25). Plasma K/T is also elevated in people living with HIV (PWH) compared to controls, especially in those who have progressed to AIDS (27, 31). While a link between K/T and Treg/Th17 ratio has been established in HIV, similar data are lacking in TB.

Here, we characterized subsets of Th17 cells defined by distinct criteria in participants with ART-suppressed HIV (HIV-ART) to determine whether IL-17–producing CD4 T cells that respond to *Mtb* antigens are altered in HIV-ART and to test the hypothesis that indoleamine 2,3-dioxygenase (IDO) activity and generation of kynurenines are associated with Th17 differentiation in HIV-ART.

## 2 Materials and methods

### 2.1 Participants and sample collection

Peripheral blood mononuclear cells (PBMC) and plasma were obtained from adults with or without HIV enrolled in the UCSF SCOPE cohort (27, 32). The participants selected for the current study were tested for sensitization to *Mtb* by tuberculin skin test (TST). All participants gave written informed consent using protocols approved by the UCSF institutional review board. Blood was collected into ethylenediaminetetraacetic acid–containing tubes for cell counts and into Acid Citrate Dextrose–containing tubes for purification of plasma and PBMC. Processed plasma was stored at −80°C, while PBMC were cryopreserved in liquid nitrogen. Demographic characteristics of the participants are in **Tables 1**, **2**. We did not have samples from all the participants in the longitudinal study (cohort 2/**Table 2**) at the start of isoniazid (INH) therapy.

**Table 1:** Cohort 1—cross sectional (characteristics of participants latent TB and with or without HIV).

Characteristic	HIV negative (*n*= 7)	HIV positive (*n*= 21)	*P*
Age, median (IQR), y	58 (44–61)	47 (38–58)	0.5395
Sex, male (%)	7 (100)	21 (100)	
Race/Ethnicity [*n*(%)]			
Black	6 (86)	8 (38)	
Asian	1 (14)	2 (10)	
White	0	7 (30)	
Middle East	0	1 (5)	
Multiracial	0	2 (10)	
Pacific Islander	0	1 (5)	
Combined antiretroviral therapy (Yes/No)	No	Yes	
CD4 counts, median (IQR), cells/mm^3^	1297 (707–1349)	640 (487–906)	0.0082
Plasma viral load (copies/mL)	None	< 40	

**Table 2:** Cohort 2—longitudinal (characteristics of participants with HIV before and after LTBI diagnosis and treatment).

Characteristic	TST neg	TST + INH initiation	TST + INH completion	*P*
Age, median (IQR), y	45 (42–51)	_	_	
Sex, male (%)	10 (71)	_	_	
Race/ethnicity [n, (%)]				
Black	5 (38)	_	_	
Asian	1 (8)	_	_	
White	6 (46)	_	_	
Pacific Islander	1 (8)	_	_	
CD4 counts, median (IQR), cells/mm^3^	527 (230–781)	442 (367–588)	464 (361–724)	0.9172
HIV infection (*n*, %)	13 (100)	11 (100)	13 (100)	
Detectable viral load [*n*; median (IQR), copies/mL]	10; 3455 (614–24888)	6; 4895 (497–35920)	6; 2980 (170–59542)	0.9632
Suppressed viral load (*n*)	3	5	7	
Combined antiretroviral therapy				
On treatment (*n*)	7	6	10	
Not on treatment (*n*)	6	5	3	

### 2.2 Plasma tryptophan and kynurenine measurement

Tryptophan and kynurenine concentrations were measured by liquid chromatography–mass spectrometry as previously described (27, 31).

### 2.3 PBMC antigen stimulation and intracellular cytokine staining

Cryopreserved PBMC were thawed in a 37°C water bath and transferred into pre-warmed R10 media (RPMI 1640 containing L-glutamine with 10% FBS, 1% PenStrep, and 1% Hepes). Cells were centrifuged at 900*g* for 5 min at room temperature, supernatants discarded, and cells resuspended before adding 5 ml of warm R10 and transferred to a 6-well culture plate and rested overnight in 37°C/5% CO_2_ incubator. After resting, 1 × 10^6^ live cells resuspended in 200 µL of R10 were transferred to each well of a 96-well round bottom plate, and duplicate wells were stimulated with *Mtb* peptide mega pool (Mtb300) (33) (2 µg/ml), cytomegalovirus (CMV) pp65 peptide (1 µg/ml) or staphylococcal enterotoxin B (SEB) positive control (1 µg/ml) in the presence of costimulating antibodies anti-CD28 (1 µg/ml) and anti-CD49d (1 µg/ml) (BD). *Mtb* peptide megapool represents commonly recognized epitopes from 90 *Mtb* antigens recognized by HLA class II–restricted CD4 T cells in diverse populations and across species (3, 34–42). CMV pp65 (PepTivator^®^CMV pp65 – Milteny Biotec, San Jose, California, USA) is a peptide pool covering the complete sequence of the pp65 protein of CMV. Negative controls received no stimulation. After 2h, GolgiStop and GolgiPlug (BD Biosciences, Franklin Lakes, New Jersey, USA) were added to one well of each of the stimulation conditions, and the cells were incubated for an additional 18h.

After 20h total stimulation, cells were washed, stained with Live/Dead Fixable Blue Dead Cell stain kit (Invitrogen, Waltham, Massachusetts, USA), washed again, then surface antibody cocktail (αCD3 BV510 clone UCHT1, 1:80; αCD8 BV570 clone RPA-T8, 1:80; αCCR7 BV785 clone G043H7, 1:20; αCD95 Alexa Fluor700 clone DX2, 1:80; αCCR6 FITC clone G034E3, 1:80; αCXCR3 BV 605 clone G025H7, 1:40; αCD161 APC-Fire750 clone HP-3G10, 1:80; αCD69 BV650 clone FN50, 1:20; αCD137 BV711 clone 4B4-1, 1:20, αOX40 PE-Cyanine7 clone Ber-ACT35, 1:40; and αTRAV1-2 (Vα7.2) BV421 clone 3C10, 1:40 (all from BioLegend, San Diego, California, USA), αCD4 BUV496 clone SK3, 1:80, αCD45RA BUV395 clone 5H9, 1:40, αCD27 BUV615 clone L128, 1:80, αCD25 BUV563 clone 2A3, 1:80, αCD39 BUV737 clone TU66, 1:80 and αCD26 BUV805 clone M-A261, 1:80 (all BD Biosciences, Franklin Lakes, New Jersey, USA) diluted in Brilliant Violet buffer (BD Biosciences, Franklin Lakes, New Jersey, USA) was added then kept in the dark for 20 min at room temperature. After washing, cells were fixed and permeabilized using eBioscience FOXP3/Transcription Factor staining kit (Invitrogen, Waltham, Massachusetts, USA). Cells were washed twice with 1X eBioscience Perm diluent, then resuspended in intracellular antibody mix (αRORγT Alexa Fluor 647 Clone Q21-559, 1:40, αIFNγ BB700 clone 2B7, 1:160 (both from BD Biosciences, Franklin Lakes, New Jersey, USA), αIL-17 PE clone BL168, 1:20, αT-bet PE-Dazzle 594 clone 4B10, 1:40 (both from BioLegend, San Diego, California, USA) and αFoxP3 PE-Cyanine5.5 clone PCH101, 1:40 (Thermo Fisher Scientific, Waltham, Massachusetts, USA) in eBiosciences perm diluent. Cells were then washed (centrifuged at 900*g* for 5 min at room temperature) twice with 1X eBioscience Perm diluent and fixed in 2% PFA. Data were acquired on a 5-Laser Aurora Spectral Flow cytometer (Cytek Biosciences, Fremont, California, USA).

### 2.4 Data and statistical analysis

Spectral flow fcs files were initially analyzed using SpectroFlo v3.0 (Cytek Biosciences, Fremont, California, USA) for unmixing and autofluorescence correction. Identification of T-cell subsets from unmixed fcs files was performed using Flowjo v10 (Flowjo LLC, Ashland, Oregon, USA). Antigen-specific cytokine levels are reported after subtraction of values of unstimulated cells for each participant. K/T ratio was determined from the concentrations of tryptophan and kynurenines after quality control checks. Non-parametric tests were used for comparison between two groups for paired (Wilcoxon test) or unpaired (Mann–Whitney test) and *p*< 0.05 was considered significant. Spearman correlation was used to test the association between parameters with a *p*< 0.05 considered significant. All statistical analyses used Prism 9.0 (GraphPad, Boston, Massachusetts, USA).

## 3 Results

### 3.1 Subsets of circulating T-helper 17 cells are disproportionately reduced in LTBI with treated HIV

**Figure 1:**
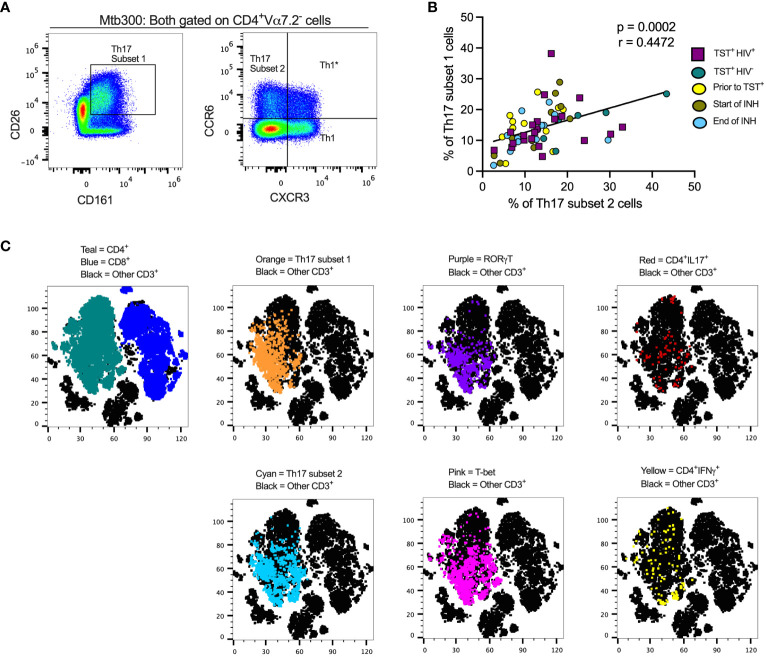
Different definitions of T helper 17 cells reveal high but incomplete concordance. **(A)** Representative flow cytometry plots and gating schemes for definition of Th17 cells by published marker criteria independent of cytokine expression. Right panel, chemokine receptor CCR6 and CXCR3 expression used to distinguish subset 2, Th1^*^, and Th1 non-MAIT CD4 T cells. Left panel: Use of surface expression of CD26 and CD161 to identify subset 1 non-MAIT CD4 T cells. Cells in both panels were stimulated with the MTB300 antigenic peptide pool. **(B)** High correlation of subset 1 (CD26^+^CD161^+^) and subset 2 (CCR6^+^CXCR3^-^) non-MAIT CD4 T cells in cell populations from participants with varying TB and HIV status. Spearman correlation p and r values are shown for results pooled from all participants. **(C)** t- stochastic neighbor embedding (t-SNE) analysis reveals incomplete concordance of transcription factor (RORγt and T-bet) and cytokine (IFNγ and IL17) production and surface phenotypes of non-MAIT CD4^+^T cells. In this analysis, protein transport inhibitors (golgi stop and golgi plug) were not added to PBMC during antigen stimulation thus cytokine detection was not optimal. Prior to TST^+^= participants were considered Mtb unexposed due to a negative TST test; INH = isoniazid therapy. Statistic: Spearman correlation.

To study the effects of LTBI and ART-treated HIV (HIV-ART) on Th17 cells defined by different criteria, we designed a spectral flow cytometry panel encompassing cytokines, chemokine receptors, transcription factors, and cell surface markers (**Supplementary Figure S1**, **Figures 1A, C**). We used the following nomenclature: (a) subset 1 is CD26^+^CD161^+^ (16, 17); (b) subset 2 is CCR6^+^CXCR3^-^ (12, 15); and (c) Th1* (Th1Th17) is CCR6^+^CXCR3^+^ (15, 43). We first gated out Vα7.2^+^CD4^+^T cells to exclude mucosal-associated invariant T (MAIT) cells (**Supplementary Figure 2**) that share certain markers with Th17 cells (44–49), and since MAITs can produce IL-17 (50).

We also stained for RORγT, the Th17 lineage defining transcription factor (51), and used unsupervised clustering and t-distributed stochastic neighbor embedding (tSNE) to determine whether subset 1 and subset 2 Th17 cells express RORγT and/or produce IL-17 upon stimulation with an *Mtb* peptide mega pool (Mtb300) (**Figure 1C**). To identify Th1* (also termed Th1Th17) cells that respond to *Mtb* antigens (15), we included T-bet, the Th1 lineage transcription factor (52). We found a positive and significant association (*r*= 0.4472, *p*= 0.0002) (**Figure 1B**) between subset 1 and subset 2 Th17 cells and found that cells in both populations express RORγT and produce IL-17 (**Figure 1C**). Therefore, subset 1 and subset 2 Th17 cells express other properties of bona fide Th17-cell populations. T-bet expressing cells were present within subset 1 and subset 2 Th17-cell populations, suggesting a Th1* population that expresses both CCR6 and CXCR3 and likely produce both IFNγ and IL-17 (**Figure 1C**). Thus, we identified the Th17 subsets based on chemokine receptors (CCR6^+^in the absence of CXCR3 expression) or surface markers (CD26 and CD161) co-expression. Since protein transport inhibitors alter the expression of some of the T cell markers (**Supplementary Figure 3**), our classification of Th17 subsets was performed on samples in which GolgiStop and GolgiPlug were not added. 

**Figure 2:**
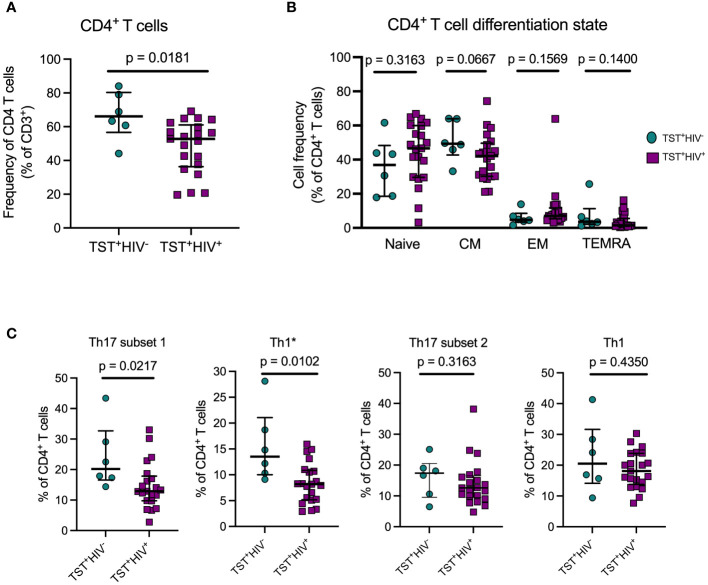
ART treated HIV infection is associated with disproportionate depletion of distinct subsets of T helper cells. **(A)** Reduced frequency of bulk CD4 T cells in HIV^+^vs HIV^-^participants in Cohort 1. **(B)** ART-treated HIV is not associated with differential distribution of CD4 T cells in memory T cell subsets. Naive: CD45RA^+^CCR7^+^; Central Memory (CM): CD45RA-CCR7^+^; Effector Memory: CD45RA^-^CCR7^-^; TEMRA: CD45RA^+^CCR7^-^. **(C)** Disproportionate reduction in frequencies of subset 1 and Th1^*^ but not subset 2 or Th1 cells in ART-treated HIV. Subset 1 = CD4^+^Vα7.2^-^CD26^+^CD161^+^; Th1^*^ (also termed Th1Th17): CD4^+^Vα7.2^-^CCR6^+^CXCR3^+^; subset 2: CD4^+^Vα7.2^-^CCR6^+^CXCR3^-^; and Th1: CD4^+^Vα7.2^-^CCR6^-^CXCR3^+^. Statistical comparisons were by Mann-Whitney test.

As expected, circulating CD4 T cells were reduced in HIV-ART compared with HIV-uninfected participants, (**Figure 2A**). We first phenotyped CD4^+^T-cell populations based on CD45RA and CCR7 (3) to identify naïve, central memory, effector memory, and terminally differentiated T cells (**Supplementary Figure S4A**, left). The predominant populations were naïve and central memory T cells and there was no difference in the frequency of cells in these memory populations in participants with or without HIV (**Figure 2B**). The expression of CD45RA and CCR7 was not affected by 20hour stimulation of PBMC with antigens (**Supplementary Figure 5**). CD4^+^CD45RA^+^CCR7^+^(naïve T cells) are a heterogeneous population, including cells that produce cytokines upon short-term stimulation (53), so we characterized the naïve T cells further based on co-expression of CD95 and CD27 to identify cells with a stem cell-like memory phenotype (**Supplementary Figure S4A**, right). We found no difference in frequencies of CD95^+^CD27^+^stem cell–like memory cells or CD95^−^CD27^+^“true naïve” cells in those with or without HIV (**Supplementary Figure S4B**). Thus, whereas individuals with treated HIV have lower overall frequencies of circulating CD4^+^T cells than those without HIV (**Table 1**, **Figure 2A**), their memory states do not differ. These observations are in accord with studies that antiretroviral therapy induces a burst in CD4 T-cell memory regeneration and general reconstitution of CD4 T-cell count relative to pre-treatment time points (54–56).

Next, we quantitated Th17 cells in those with LTBI without HIV or with HIV-ART and found significantly lower frequencies of subset 1 (CD4^+^Vα7.2^−^CD26^+^CD161^+^) and Th1* (CD4^+^Vα7.2^−^CCR6^+^CXCR3^+^) cells (expressed as % of CD4^+^ T cells) in HIV-ART than in those without HIV (**Figure 2C**). Although subset 2 cells (CD4^+^Vα7.2^−^CCR6^+^CXCR3^−^) and Th1 (CD4^+^Vα7.2^−^CCR6^−^CXCR3^+^) cells were also present at lower frequencies in HIV-ART, the difference was not significant (**Figure 2C**). These data demonstrate that CD26^+^CD161^+^(subset 1) and CCR6^+^CXCR3^+^ (Th1*) circulating Th17 cells are disproportionately depleted in HIV-ART, while CCR6^+^(subset 2) Th17 cells and Th1 (CCR6^-^CXCR3^+^) cells are relatively preserved.

Th17 and regulatory T cells (Tregs) develop under related but distinct cytokine environments (57). Tregs contribute to immune homeostasis by maintaining unresponsiveness to self-antigens, suppressing exaggerated immune responses, and promoting epithelial tissue integrity (58). Human Tregs are identified by the expression of high levels of CD25 and the transcription factor FoxP3 (59). Since the kynurenine pathway has differential effects on Th17 and Treg differentiation, we characterized Treg cells (**Supplementary Figure S6A**, left) and found that the frequency of circulating Treg was similar in HIV-ART and those without HIV (**Supplementary Figure S6B**). We also found that the frequency of activated (CD39^+^) Tregs did not differ in the two groups (**Supplementary Figure S6A**, right), although there was considerable variation in CD39^+^ Treg, especially in HIV-ART (**Supplementary Figure S6C**). We also compared the ratio of Th17 subset 1 and Th17 subset 2 cells to Tregs between the groups and found a trend toward a lower Th17 subset1/Treg in HIV-ART than in those without HIV (**Supplementary Figure S6D**), but the difference was not significant, and Th17 subset 2/Treg ratios were similar in HIV-ART and those without HIV (**Supplementary Figure S6E**). Taken together, we found that CD26^+^CD161^+^ Th17-cell subset is disproportionately reduced in LTBI with treated HIV, suggesting that even effective treatment of HIV does not reconstitute all Th17-cell subsets equally. We did not find differences in Treg by HIV status.

### 3.2 Mtb antigen-responsive IL-17–producing cells are enriched in subset 1 compared with subset 2 Th17 cell populations in HIV-ART

**Figure 3:**
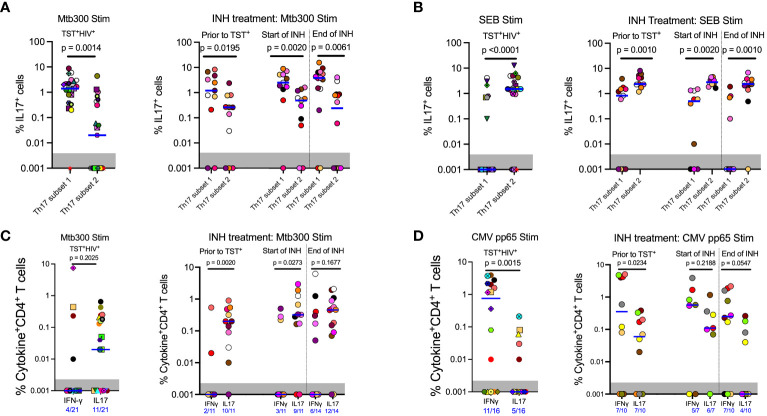
In contrast to responses to other stimuli, *Mtb* antigen responsive IL17 expression is enriched in subset 1 (CD26^+^CD161^+^) rather than subset 2 (CCR6^+^CXCR3^-^) Th17 cells. **(A)** Left panel (Cohort 1): CD4 T cells that produce IL17 in response to MTB300 are predominantly in subset 1. Right panel (Cohort 2): MTB300-stimulated IL17 production predominates in subset 1 rather than subset 2 cells regardless of TST status and is not altered by INH preventive therapy. **(B)** Left panel (Cohort 1): In contrast to MTB300-responsive cells, IL17 production predominates in subset 2 cells in response to SEB stimulation. Right panel (Cohort 2): INH preventive therapy does not alter the pattern of IL17 production by SEB-stimulated subset 2 or subset 1 Th17 cells. **(C)** Left panel (Cohort 1): In ART-treated HIV participants, CD4 T cells that produce IFNγ in response to MTB300 stimulation are present at low frequencies. Right panel (Cohort 2): **(D)** Left panel (Cohort 1): CMV pp56 antigen stimulation induces higher frequencies of IFNγ than IL17-producing CD4 T cells. Right panel (Cohort 2): INH treatment does not alter CD4 T cell responses to CMV pp65 antigen stimulation. Statistics: Wilcoxon test.

Having established that subset 1 (CD26^+^CD161^+^) and subset 2 (CCR6^+^) Th17 cells are bona fide Th17 populations, we determined whether subset 1 and subset 2 cells differ in IL-17 production after *Mtb* antigen stimulation. HIV-ART participants with LTBI had significantly more IL-17^+^cells in subset 1 Th17 cells than in subset 2 Th17 cells upon stimulation with the Mtb300 peptide pool (**Figure 3A**, left). All except one participant had IL-17^+^cells in subset 1 cells compared with 11 of 21 participants with IL-17^+^cells in subset 2 Th17 cells (**Figure 3A**, left). We did not have enough cells from participants without HIV (cohort 1) for cytokine analysis. We confirmed this observation in cohort 2; regardless of the sampling time point, there were significantly more IL-17^+^cells in subset 1 Th17 cells than in subset 2 Th17 cells (**Figure 3A**, right). We performed a similar analysis after stimulation with SEB and found an opposite pattern: IL-17 production was significantly higher in subset 2 than in subset 1 Th17 cells (**Figure 3B**). Additionally, CMV-peptide stimulation induced equivalent production of IL-17 by subset 1 and subset 2 Th17 cells, although there was a trend toward higher responses in subset 2 Th17 cells (**Supplementary Figure S7A**). These data reveal enrichment of IL-17 production to *Mtb* antigens by a subset of Th17 cells marked by expression of CD26 and CD161 (subset 1), which are distinct from IL-17–producing cells that are marked by expression of chemokine receptor CCR6 (subset 2) and those that produce IL-17 in response to SEB or CMV (**Supplementary Table 1**).

### 3.3 Mtb antigen–responsive IL-17–producing CD4^+^ T cells are relatively preserved in people with LTBI and HIV-ART

Next, we assessed IFNγ and IL-17 production after Mtb300 stimulation. In HIV-ART, only four of 21 (19%) had detectable IFNγ responses compared with 11 of 21 (52%) who had detectable IL-17 responses (*p*= 0.0516, Fisher’s exact test). Additionally, the median IL-17 response was higher than the median IFNγ response, although this did not reach significance (**Figure 3C**, left). Similar IL-17 responses to *Mtb* antigen stimulation were observed in people with HIV before and after LTBI diagnosis and treatment. Treatment of LTBI did not change either IFNγ or IL-17 responses, although the response magnitudes varied by participant (**Figure 3C**, right). Similarly, more participants had detectable IL-17 responses than detectable IFNγ responses at all three sampling times, prior to being TST positive, and at INH initiation and treatment completion: 2/11 versus 10/11, 3/11 versus 9/11, and 6/14 versus 12/14, respectively (**Figure 3C**, right).

To determine whether the predominant production of IL-17 over IFNγ was specific to *Mtb* antigens, we used a CMV peptide pool to stimulate PBMC from participants (**Tables 1**, **2**) who had sufficient cells (**Figure 3D**). This revealed a pattern distinct from that with Mtb300: participants with LTBI and HIV-ART (cohort 1) exhibited higher magnitude and higher prevalence of IFNγ than IL-17 responses [IFNγ: 11/16 (69%) vs. IL-17: 5/16 (31%)] (**Figure 3D**, left) (*p*= 0.0756, Fisher’s exact test). Similarly, in Cohort 2, there was a higher magnitude of IFNγ responses than IL-17 responses at all time points, reaching statistical significance at baseline (prior to TST^+^) time point (**Figure 3D**, right). As with CMV and in contrast to Mtb300, SEB induced higher magnitude IFNγ than IL-17 responses (**Supplementary Figure S7B**). Together, these data demonstrate that in LTBI and HIV-ART, IL-17 responses to *Mtb* are relatively preserved compared with IFNγ responses, and this is distinct compared to CMV and SEB responses. This is also contrary to the commonly held impression that IFNγ production is the predominant response to *Mtb*.

### 3.4 IDO activity is increased in people with LTBI and HIV-ART, and negatively correlates with circulating Th17 cells

**Figure 4:**
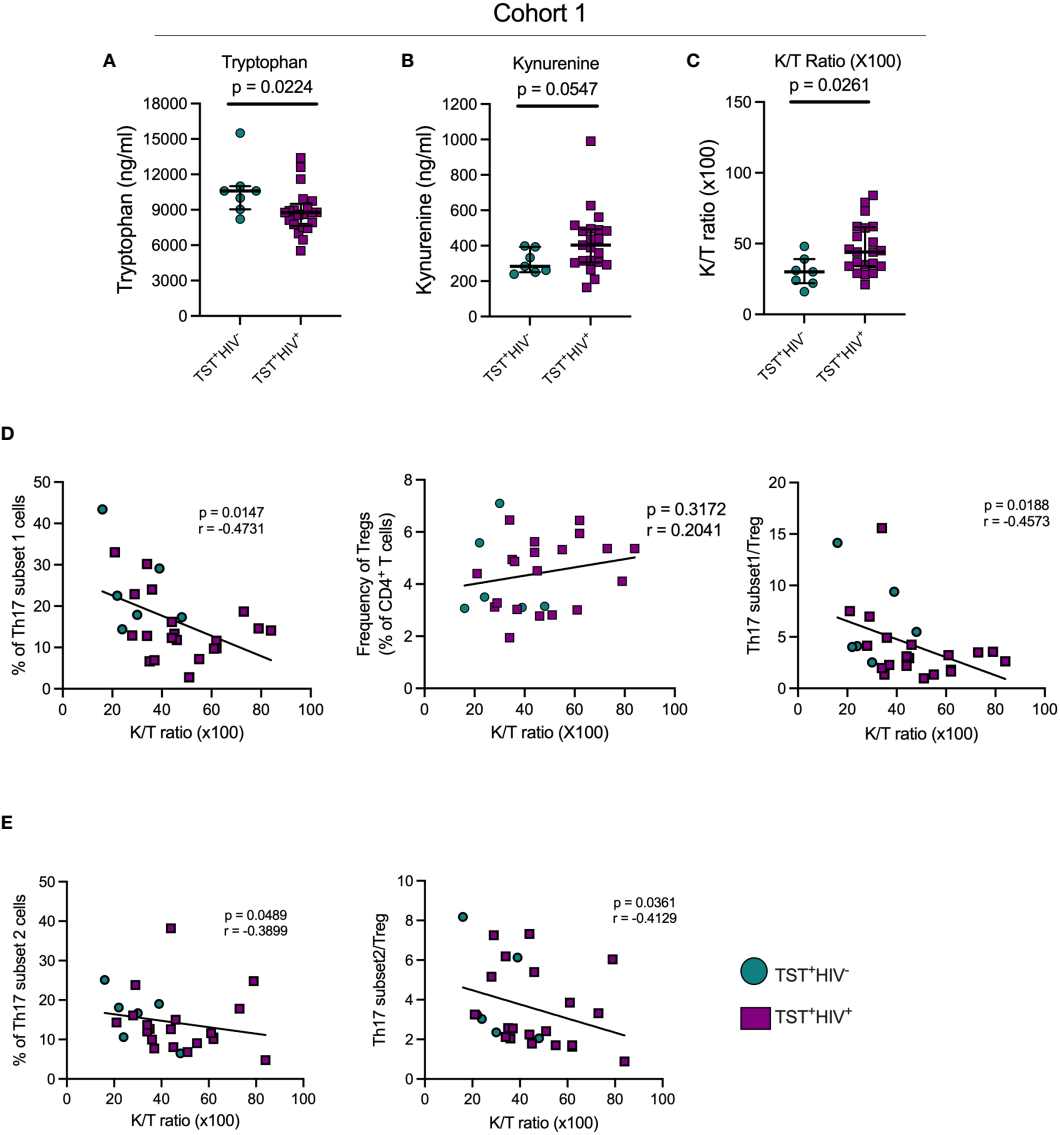
In TST^+^participants, HIV infection is associated with increased indoleamine-2,3-dioxygenase (IDO) activity, which negatively correlates with Th17 cell subsets. **(A)** Plasma tryptophan concentrations; **(B)** Plasma kynurenine concentrations; **(C)** Plasma Kynurenine (K)/Tryptophan (T) ratios as a reflection of IDO activity. **(D)** Left panel: correlation of subset 1 Th17 cell frequencies with plasma K/T ratios; Middle panel: Correlation of regulatory T cells and plasma K/T ratios; Right panel: Correlation of the ratio of subset 1/Treg ratios and plasma K/T ratios. **(E)** Left panel: correlation of subset 2 Th17 cell frequencies with plasma K/T ratio; Right panel: Correlation of the ratio of subset 2/Treg ratios and plasma K/T ratios. Statistical analyses were by Mann-Whitney **(A–C)** or Spearman correlation **(D, E)**.

We quantitated tryptophan and kynurenine concentrations in plasma of participants with LTBI with or without HIV-ART and inferred IDO activity based on the K/T ratio. We found significantly lower plasma tryptophan concentrations in participants with HIV-ART compared with those without HIV (**Figure 4A**), and kynurenine concentrations were higher in HIV-ART but did not achieve statistical significance when compared with the HIV uninfected group (**Figure 4B**). Finally, there was a significantly higher K/T ratio in people with HIV-ART compared to those without HIV (**Figure 4C**). Although we cannot ascertain the contribution of LTBI to the observed IDO activity because we did not have participants who were not exposed to *Mtb*, these observations agree with prior reports showing that IDO-1 mediated tryptophan catabolism is elevated in PWH, including those initiated on early treatment and ART-suppressed viral loads (31).

Since kynurenines favor the development of Tregs while suppressing Th17 generation (27), we determined the correlation of plasma K/T and Th17 cells, Tregs, and Th17/Treg ratios in cohort 1 participants. This revealed a significant inverse relationship between K/T and circulating subset 1 and subset 2 (**Figures 4D, E**), while there was no correlation between K/T and Tregs (**Figure 4D**, middle). We also found significant inverse correlations between K/T and subset 1/Treg and subset 2/Treg (**Figures 4D, E**). Importantly, we found no correlation between K/T and Th1*, Th1, or IL-17^+^cell frequencies (**Supplementary Figures S8A**, **S8B** and data not shown). Although we confirmed high IDO activity (**Figure 4**) and found a reduction in circulating Th17 (especially subset 1) cells in HIV-ART compared to HIV uninfected people (**Figure 2**), Tregs and the Th17/Treg ratio were similar in the two groups (**Supplementary Figure S3**). Thus, increased IDO activity is associated with a reduction in circulating Th17 cell subsets, but the frequency of IL-17–producing CD4 T cells does not correlate with IDO activity.

## 4 Discussion

The protective role of IL-17–producing CD4 T cells against *Mtb* infection has been demonstrated across species: mice (60–62), non-human primates (63–65), and humans (1–3). Studies that demonstrate Th17 cells contribute to the control of *Mtb* infection in humans used different approaches to identify the cells. We used a combination of cell markers (12, 15–17, 20, 21) to broadly characterize Th17 cells and then confirmed that they produce IL-17, instead of using IL-17 production alone to define Th17 cells (3, 66). Since there are cytokine-independent mechanisms of T cell responses, and since cytokine production can be influenced by stimulation conditions, our approach affords a broader characterization of Th17 cells to determine the impact of HIV coinfection. We also considered other available techniques for studying Th17 cells such as sequencing approaches. Our approach of first identifying Th17 cells based on cell surface markers is compatible with single-cell sequencing that has revolutionized the study of CD4 T cells beyond cytokine production. The existing technologies do not reliably allow the sequencing of cells after fixation and permeabilization for the detection of cytokines.

A recent study in macaques showed granulomas that controlled *Mtb* were enriched for Th1Th17 (Th1*) cells that did not express IL-17 transcripts (65). Furthermore, since *Mtb* antigen–specific cells must traffic to sites of disease, using chemokine receptor expression alone in blood may underreport the population of these cells. Our finding of disproportionate reduction of specific circulating Th17 cell categories in HIV-ART reveals the importance of using multiple criteria to identify CD4 T cells of interest, and that ART-mediated suppression of HIV does not reconstitute all Th17 cell types equally. Previous reports indicate that CD4^+^CD161^+^ T cells represent a subset of fast acting T cells that inhibit mycobacterial growth in unexposed humans but not TB patients (67) and are also significantly reduced in active TB compared to latent TB (68), suggesting their protective immunity against *Mtb* infection. Although these studies did not include CD26, they may probably constitute subset 1 Th17 cells identified in our study. The preferential production of IL-17 by Th17 subsets 1 and 2 depending on the type of stimulation reiterates the need to study pathogen-specific responses using the antigens specific to the pathogen of interest, and that responses to polyclonal stimulation with superantigens may overestimate responses by distinct T-cell subsets. Studies to identify CD4 T-cell defects that contribute to TB susceptibility in HIV and HIV-ART will benefit from higher resolution characterization of CD4^+^ T-cell subsets.

Our finding of a higher magnitude of IL-17 than IFNγ-producing CD4^+^ T cells in HIV-ART upon Mtb300 stimulation is contrary to the impression that IFNγ production is the dominant response to *Mtb* infection (69) but agrees with a recent study that revealed evidence that people who have been exposed to *Mtb* but remain negative by IFNγ responses have detectable responses to *Mtb* antigens when other readouts are used (70). Until recently, those individuals were thought to remain uninfected by *Mtb*; our results add weight to the evolving concept that considering IFNγ as the canonical response to *Mtb* infection requires reconsideration.

Although the Mtb300 peptide megapool that we used in our study contains peptides from multiple *Mtb* antigens including ESAT6 and CFP10, the sensitivity of our assay may be lower compared to the IGRA test because QuantiFERON-TB Gold Plus blood test is designed to detect both CD4 and CD8 T cells and uses whole blood; we only report on CD4^+^ T cells in PBMC. Our findings indicate that IL-17 responses to *Mtb* but not CMV antigens or SEB can be preserved in HIV-ART. We hypothesize that IL-17–producing cells may be less activated than IFNγ-producing cells and/or express the CCR5 coreceptor at lower levels or at lower frequencies and are thus preserved from HIV infection. The technical challenge of optimal measurement of T-cell activation and cytokine production in one condition (detection of cytokines requires protein transport inhibitors which hinder T-cell activation marker expression) precluded us from testing this hypothesis.

The K/T ratio is a strong predictor of mortality risk in ART-suppressed PWH (71), does not normalize in PWH initiating ART early, and durably suppressed early ART initiators have high plasma KT ratios compared with HIV-negative controls (31). Therefore, the elevated tryptophan catabolism in HIV-ART reported here and previously (27, 31) may explain, in part, the persistently increased risk of tuberculosis in treated HIV. Inhibition of IDO-1 with 1-methyl-D-tryptophan (indoximod) in macaques was reported to decrease *Mtb* burdens and pathology, and increased the proliferation of CD4^+^ and CD8^+^memory T cells (72). Together, these results suggest that enhancing Th17 cell responses by IDO inhibition may be beneficial in the context of TB.

Our study has limitations: (1) the participants were from a study investigating the long-term clinical and immunological consequences of HIV infections and their treatment; therefore, we had few HIV-uninfected participants; (2) 10 of 13 PWH initially with a negative TST prior to TST conversion had high viral loads that probably reduced the sensitivity of diagnosis; (3) INH treatment was not given as directly observed therapy (DOT), and we could not ascertain treatment compliance by the study participants. Nevertheless, we found that Th17 cells are a heterogenous population and IL-17 responses to *Mtb* are preserved in HIV-TB–coinfected individuals, and we demonstrated the link between IDO activity and circulating *Mtb*-responsive Th17 cells in humans. With IDO-1 inhibitors in clinical trials (73), management of TB disease might benefit from simultaneous therapeutic inhibition of IDO-1 activity to enhance Th17 cell responses, particularly in PWH where HIV further enhances tryptophan catabolism via the kynurenine pathway.

## Data availability statement

The original contributions presented in the study are included in the article/**Supplementary Material**. Further inquiries can be directed to the corresponding author.

## Ethics statement

The studies involving humans were approved by UCSF Institutional Review Board. The studies were conducted in accordance with the local legislation and institutional requirements. The participants provided their written informed consent to participate in this study.

## Author contributions

PO: Conceptualization, Data curation, Formal analysis, Funding acquisition, Investigation, Methodology, Writing – original draft, Writing – review & editing. AT: Data curation, Methodology, Writing – review & editing. FM: Data curation, Methodology, Writing – review & editing. DG: Data curation, Methodology, Writing – review & editing. MK: Data curation, Resources, Writing – review & editing. FA: Data curation, Methodology, Writing – review & editing. CSLA: Resources, Writing – review & editing. JNM: Resources, Writing – review & editing. SGD: Resources, Writing – review & editing. PWH: Conceptualization, Funding acquisition, Resources, Supervision, Writing – review & editing. JDE: Conceptualization, Funding acquisition, Resources, Supervision, Writing – review & editing.
