# Supplementary material for: High-parameter phenotypic characterization reveals a subset of human Th17 cells that preferentially produce IL-17 against M. tuberculosis antigen
Source: Front Immunol. 2024 Apr 18;15:1378040. doi: 10.3389/fimmu.2024.1378040 (PMC11064812; doi:10.3389/fimmu.2024.1378040)
Supplement: Supplementary file 2 [file Table_1.pdf]

Supplemental Table 1. Frequencies of Th17 cell subsets in antigen-specific $CD4^+IL17^+$ cells

	Median % (interquartile range)			p value (Kruskal-Wallis)
	**Th17 Subset1**	**Th17 Subset2**	**Th1* (Th1/Th17)**	
Mtb300 Stimulated (n=35)	30.1 (19.8, 37.1)	26.1 (18.1, 38.2)	15.1 (9.9, 18.9)	<0.0001
CMVpp65 Stimulated (n=30)	24.1 (15.8, 33)	26.5 (19, 35.6)	10.6 (6.4, 16.4)	<0.0001

Protein transport inhibitors was added to PBMC 2 hours after the start of antigen stimulation (for optimal detection of IL17) and stimulation was allowed for additional 18 hours followed by intracellular cytokine detection. For this analysis, only samples with ≥ 100 $CD4^+IL17^+$ cells were used to subset $IL17^+$ population into Th17 subsets: Subset1 ($CD4^+V\alpha7.2^-CD26^+CD161^+$), Subset2 ($CD4^+V\alpha7.2^-CCR6^+CXCR3^-$) and Th1* (Th1Th17 ($CD4^+Va7.2^-CCR6^+CXCR3^+$))
